# Periprocedural Bridging Therapy in Patients With Mechanical Heart Valves

**DOI:** 10.7759/cureus.56465

**Published:** 2024-03-19

**Authors:** Nivedha Balaji, Oluwafemi Olukayode, Fardeen Faiz, Priyadarshini Dixit, Vedang Bhavsar

**Affiliations:** 1 Internal Medicine, Northeast Georgia Medical Center Gainesville, Gainesville, USA; 2 Cardiology, Northeast Georgia Medical Center Gainesville, Gainesville, USA

**Keywords:** low-molecular-weight heparin, bridging anticoagulation, thrombus, unfractionated heparin, mechanical heart valves

## Abstract

Mechanical heart valves (MHVs) are thrombogenic and require lifelong anticoagulation with vitamin K antagonists (VKAs) such as warfarin. Periprocedural bridging with unfractionated heparin (UFH) and low-molecular-weight heparin (LMWH) aims to reduce the risk of thromboembolic events in patients. Currently, there are no definitive class I recommendations for anticoagulation management in patients with MHVs. In this report, we present the case of a 77-year-old female who was perioperatively bridged with enoxaparin and subsequently developed an acute thrombus.

## Introduction

Valvular heart disease impacts approximately 2.5% of the US population; mitral and aortic valve diseases are the most common valvular diseases. There has been a nationwide preference for bioprosthetic over mechanical valves due to the need for lifelong anticoagulation with mechanical valves [1]. Mechanical prosthetic valves are thrombogenic and carry a risk of cerebrovascular accident, systemic embolism, and prosthetic valve thrombosis [2]. The annual incidence of mechanical valve thrombosis is estimated to range from 0.5% to 0.8% for mitral and aortic prostheses and 20% for tricuspid prostheses [3]. However, the incidence is even higher within the first three months of implantation [2]. The annual risk for thromboembolism in patients without anticoagulation and with mechanical heart valves (MHVs) is estimated to be around 8%, but this risk is decreased by 80% with warfarin [4]. Therefore, mechanical prostheses require strict compliance with lifelong anticoagulation [1,2,5]. Annually, approximately 10% of patients or over 250,000 individuals in North America receiving vitamin K antagonist (VKA) may require interruption in anticoagulation therapy for invasive or surgical procedures [6]. We present to you the case of a 77-year-old female with a medical history of mitral valve stenosis secondary to rheumatic fever status post mechanical mitral valve replacement (MVR) and recent L3-L5 laminectomy and foraminotomy who underwent bridging therapy with enoxaparin. 

## Case presentation

A 77-year-old female with a medical history of mitral valve stenosis due to rheumatic fever status post mechanical valve replacement in 2012 and recent bridging therapy with enoxaparin for L3-L5 laminectomy and foraminotomy presented to the emergency department (ED) nine days post-procedure for acute and progressively worsening shortness of breath. 

Of note, prior to the procedure, the patient had seen a physician at the pre-surgical clinic and was advised to hold warfarin five days prior to surgery and bridge with enoxaparin 1 mL of 100 mg/mL of enoxaparin every 12 hours once her international normalized ratio (INR) dropped below 2.0. At the time of the visit, her INR was 3.14. During this time, her anti-Xa levels were not checked. The patient was given a prescription for six days' worth of enoxaparin and had completed her prescription. At this point, the patient mentioned that she had run out of supplies for testing INR at home and that she was unable to visit a physician's office in a timely manner for refills. However, her surgery was postponed, and due to a delay in follow-up on the patient's part, she missed two days' worth of bridging therapy. 

The patient was discharged on postoperative day 8 and was asked to restart warfarin after postoperative day 10. Of note, she returned to the ED on the same day with complaints of low back pain and was discharged from the ED with oral pain medication. Unfortunately, she returned to the ED the following day with complaints of shortness of breath. The patient was subsequently diagnosed with acute pulmonary edema causing acute hypoxemic respiratory failure in the setting of acute heart failure requiring supplemental oxygen via nasal cannula. She had denied any fevers or chills. Chest radiography noted increased interstitial opacities and pulmonary vascular markings. Her laboratory findings were significant for N-terminal pro-B-type natriuretic peptide 4740 pg/mL, high-sensitivity troponin 67 ng/L, and white blood cells 17,000 K/uL with a left shift. The patient was initiated on empiric antibiotics with intravenous vancomycin, cefepime, and azithromycin. The following day, she was restarted on bridging therapy with enoxaparin 1 mg/kg every 12 hours and warfarin with plans to continue bridging therapy until INR was therapeutic. Transthoracic echocardiography (TTE) noted mildly reduced left ventricular systolic function with an estimated ejection fraction (EF) of 45% with an elevated prosthetic gradient and mild regurgitation. Prior transesophageal echocardiography (TEE) four months earlier was remarkable for EF 50-55%, mild mitral regurgitation, and normal functioning mitral valve. Due to the newly discovered findings on the TTE and after discussion with the on-call cardiologist, the patient was transferred to another hospital facility for a higher level of care. 

**Figure 1:**
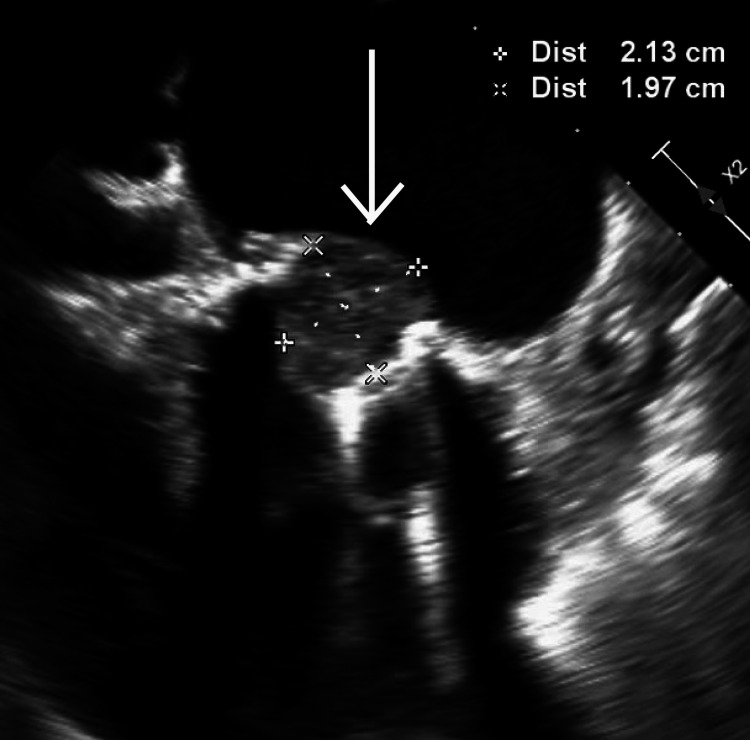
Transesophageal echocardiogram showing 2×2 cm thrombus on the left atrial side of the mechanical mitral valve

Subsequently, she was initiated on an unfractionated heparin (UFH) drip. She then had a TEE that noted severe mitral valve stenosis with an elevated prosthetic gradient of 16 mmHg and a 2×2 cm left atrial thrombus on the side of the MHV shown in Video 1 and Figure 1.

**Video 1:** Transesophageal echocardiogram showing mechanical mitral valve thrombus with severe stenosis (echogenic mass suggestive of thrombus)

The next day, on postoperative day 12, cardiothoracic surgery administered tissue plasminogen activator (tPA), and a repeat TTE noted an EF of 35-40% with a mean mitral valve gradient of 7 mmHg. On postoperative day 15, she had a TEE that showed EF 50-55% with a mitral valve gradient of 3 mmHg, large mobile mass with central lucency, consistent with a thrombus, that appeared decreased in size and attached to the suture ring of the mechanical valve leaflet. The patient then mentioned improvement in shortness of breath and was transitioned to room air after receiving tPA. Subsequently, the UFH drip was discontinued, and she was transitioned to warfarin to maintain an INR of 2.5-3.5. 

Unfortunately, on postoperative day 20, she endorsed serosanguinous secretions from the neurosurgical site. A 4x4 cm dressing was placed on the surgical site. The surgical site was examined by the neurosurgical team and was noted to be well-approximated. The bleeding was self-limiting and required no further intervention. However, she then developed bilateral lower extremity paresthesia and weakness for which she had a lumbar spine magnetic resonance imaging (MRI) that showed a large fluid collection in the postoperative site resulting in severe thecal sac effacement of L3-L5 consistent with a hematoma. Despite the MRI findings, due to the acute heart failure and the mechanical valve thrombus, the neurosurgical team postponed any further surgical intervention at that time. 

## Discussion

The etiopathogenesis of prosthetic valve thrombus formation includes (1) the absorption of plasma proteins on the prosthesis and subsequent invasion of platelets, (2) the effect of transprosthetic blood flow, (3) ineffective anticoagulation with subtherapeutic INR, and (4) other prothrombotic factors such as atrial fibrillation, multiple valve replacement, left atrial enlargement, malignancy, systemic diseases, medication-induced contraceptives, and presence of specific antibodies such as anticardiolipin and anti-tPA [3]. The target INR for patients with MHV is 2.5, but the target is increased to INR 3 if they have additional thromboembolism risks such as atrial fibrillation, left ventricular EF <35%, previous thromboembolism, hypercoagulable state, older-generation mechanical valves, left ventricular systolic dysfunction, or greater than one mechanical valve [4,7]. 

The American Heart Association (AHA)/American College of Cardiology (ACC) recommends continuation of VKA anticoagulation with a therapeutic INR for patients undergoing minor procedures where bleeding is easily controlled. They state that temporary interruption of VKA anticoagulation without bridging agents while INR is subtherapeutic is recommended for patients with bileaflet mechanical aortic valve replacement (AVR) undergoing invasive or surgical procedures but have no other risk factors for thrombosis. Bridging therapy is reasonable when INR is subtherapeutic for patients undergoing invasive or surgical procedures if they have a mechanical AVR and risk factors for thromboembolic events, have older-generation mechanical AVR, or have a mechanical MVR. Bridging therapy can include intravenous UFH or low-molecular-weight heparin (LMWH) [8,9]. However, these guidelines are based on Grade 2C evidence which is based on the weakest evidence available. 

Bridging therapy is estimated to increase the risk of perioperative bleeding by 4-8% and is correlated directly to the INR [9]. Both LMWH and UFH are distinct strategies that reduce the risk of thromboembolic events. UFH bridging therapy is administered intravenously according to a nomogram requiring perioperative hospitalization and continuous monitoring of activated partial thromboplastin time (aPTT) and anti-factor Xa, whereas LMWH can be self-administered subcutaneously without the need for daily laboratory monitoring. Additionally, LMWH has a better safety profile with a lower risk for thrombocytopenia and bleeding. UFH should be discontinued four hours prior to the procedure, whereas LMWH should be discontinued 12 hours before the surgery. However, the use of LMWH is contraindicated in patients with several renal dysfunction. Additionally, its use is limited in patients who have MHV due to the lack of randomized controlled trials and in obese individuals due to the lack of data on the optimal dosing and monitoring protocols [4]. 

PERIOP2, a randomized, double-blind, placebo-controlled trial, concluded that postoperative bridging with dalteparin was not found to be beneficial in preventing thromboembolic events in patients with MHVs [10]. The REGIMEN study, a multicenter, prospective, observational study across 14 different centers in the United States and Canada, concluded that the adverse events of thromboembolism, major bleeds, and death between bridging with LMWH and UFH remain similar [11]. Hart et al. report a retrospective multicenter study in the Netherlands that concluded that there was no difference in the rate of thromboembolism between UFH and LMWH bridging therapy [12]. While these previous studies have shown LMWH to be non-inferior to UFH for bridging therapy perioperatively, we report the case of this patient who developed an acute thrombus post-bridging with enoxaparin. However, this patient did miss two days of preoperative bridging with enoxaparin. This case report reiterates the need for perioperative bridging therapy in patients receiving chronic oral anticoagulation for MHVs. Additionally, it highlights the limitations in current guidelines and the need for high-quality, randomized controlled trials to determine the role of UFH and LMWH in perioperative anticoagulation. 

## Conclusions

It is imperative to continue bridging with anticoagulants perioperatively in patients with MHVs who are on chronic oral anticoagulation. LMWH is a more convenient means of bridging anticoagulation than UFH as UFH requires daily laboratory monitoring, but we present to you the case of this patient who developed a left atrial thrombus status post anticoagulation with LMWH. Further randomized trials are needed to compare the role of LMWH and UFH in periprocedural bridging. 
